# Gastrointestinal Endogenous Proteins as a Source of Bioactive Peptides - An *In Silico* Study

**DOI:** 10.1371/journal.pone.0098922

**Published:** 2014-06-05

**Authors:** Lakshmi A. Dave, Carlos A. Montoya, Shane M. Rutherfurd, Paul J. Moughan

**Affiliations:** Riddet Institute, Massey University, Palmerston North, New Zealand; University of South Florida College of Medicine, United States of America

## Abstract

Dietary proteins are known to contain bioactive peptides that are released during digestion. Endogenous proteins secreted into the gastrointestinal tract represent a quantitatively greater supply of protein to the gut lumen than those of dietary origin. Many of these endogenous proteins are digested in the gastrointestinal tract but the possibility that these are also a source of bioactive peptides has not been considered. An *in silico* prediction method was used to test if bioactive peptides could be derived from the gastrointestinal digestion of gut endogenous proteins. Twenty six gut endogenous proteins and seven dietary proteins were evaluated. The peptides present after gastric and intestinal digestion were predicted based on the amino acid sequence of the proteins and the known specificities of the major gastrointestinal proteases. The predicted resultant peptides possessing amino acid sequences identical to those of known bioactive peptides were identified. After gastrointestinal digestion (based on the *in silico* simulation), the total number of bioactive peptides predicted to be released ranged from 1 (gliadin) to 55 (myosin) for the selected dietary proteins and from 1 (secretin) to 39 (mucin-5AC) for the selected gut endogenous proteins. Within the intact proteins and after simulated gastrointestinal digestion, angiotensin converting enzyme (ACE)-inhibitory peptide sequences were the most frequently observed in both the dietary and endogenous proteins. Among the dietary proteins, after *in silico* simulated gastrointestinal digestion, myosin was found to have the highest number of ACE-inhibitory peptide sequences (49 peptides), while for the gut endogenous proteins, mucin-5AC had the greatest number of ACE-inhibitory peptide sequences (38 peptides). Gut endogenous proteins may be an important source of bioactive peptides in the gut particularly since gut endogenous proteins represent a quantitatively large and consistent source of protein.

## Introduction

The main role of dietary proteins is to provide amino acids for body protein synthesis [1]. However, investigations over the last two decades have shown that dietary protein can also be a source of latent bioactive peptides (from 2 to greater than 40 amino acids long) that when released during digestion in the gastrointestinal tract can act as modulators of various physiological functions [2], [3], [4]. These peptides are reported to possess a range of effects including antihypertensive, cholesterol-lowering, antioxidant, anticancer, immunomodulatory, antimicrobial, opioid, antiobesity and mineral binding effects [2], [5], [6]. The most extensively studied dietary sources of these bioactive peptides include milk, egg, meat, soya and cereal proteins [2], [3], [7], [8]. The bioactive peptides released during the digestion of dietary proteins are believed to act either within the gastrointestinal tract or are possibly absorbed into the bloodstream where they may act systemically [3], [9], [10], [11].

The supply of dietary proteins, and therefore the supply of gastrointestinal bioactive peptides derived from those proteins, will likely be highly variable as humans do not consume the same foods or amounts of food on a day to day basis. However, a considerable amount of endogenous (non-dietary) protein is also present in the lumen of the gastrointestinal tract during digestion, consisting of proteins such as mucins, serum albumin, digestive enzymes, protein within sloughed epithelial cells and microbial protein, and this material may be a source of bioactive peptides [12]. When compared to dietary protein, gut endogenous proteins represent a larger and more constant supply of protein in the gastrointestinal tract [13], [14], [15], [16], with endogenous nitrogen entering the digestive tract of humans being quantitatively equal or greater than the dietary nitrogen intake [16], [17], [18], [19], [20]. In a study conducted using pigs fed a casein-based diet, it has been reported that up to 80% of endogenous proteins are digested and reabsorbed by the end of the small intestine [14]. During digestion a wide range of endogenous protein-derived peptides are likely to be generated, but the biological activity of such endogenously sourced gut peptides has not yet been considered. Potentially, gut endogenous proteins could be an important source of gut bioactive peptides given the amount of endogenous proteins present in the gastrointestinal tract. This study aimed to use an *in silico* approach to investigate whether known bioactive peptide sequences are present within the amino acid sequences of endogenous proteins secreted along the gastrointestinal tract and whether these bioactive peptides may potentially be released during enzymatic digestion in the human gastrointestinal tract. To our knowledge, the present study is the first to show that the amino acid sequences of gut endogenous proteins hold within them abundant bioactive peptide sequences and that the possibility exists that these peptides are released during gastrointestinal digestion.

## Methods

Twenty six known human gut endogenous proteins with well characterised amino acid sequences were examined. Additionally, 7 dietary proteins, which have been reported to contain bioactive peptides, were also examined [2], [21],[22],[23],[24]. The proteins analysed are shown in Table 1.

**Table 1:** Gut endogenous and dietary proteins examined in the *in silico* study^1^.

Protein/peptide classified based on thefunction in the body, with accession number^1^	Site of secretion withingastrointestinal tract	Chain length of mature protein^2^(No. of amino acid residues)
Lubrication, maintenance of integrity oftissue lining, cell signaling, immunity		
Mucin-2 (Q02817)	Small intestine and colon	5159
Mucin-3A (Q02505)	Small intestine	2520
Mucin-3B (Q9H195)	Small intestine and colon	901
Mucin-5AC (P98088)	Stomach, oesophagus andproximal duodenum	5003
Mucin-6 (Q6W4X9)	Stomach	2417
Mucin-7 (Q8TAX7)	Salivary gland -mouth	355
Mucin-13 (Q9H3R2)	Stomach, small intestineand colon	493
Mucin-15 (Q8N387)	Small intestine and colon	311
Mucin-20 (Q8N307)	Throughout the gut	684
Maintenance of colloid osmotic pressureand acid-base balance and transport		
Serum albumin (P02768)	From plasma intostomach and intestine	591
Enzymes in digestion		
Chymotrypsinogen B (P17538)	Pancreas	245
Chymotrypsinogen B_2_ (Q6GPI1)	Pancreas	245
Gastric triacylglycerol lipase (P07098)	Stomach	379
Pancreatic amylase (P04746)	Pancreas	496
Pancreatic triacylglycerol lipase (P16233)	Pancreas	449
Pepsin (P00790)	Stomach	373
Salivary amylase (P04745)	From salivary glandinto mouth	496
Trypsin (P07477)	Pancreas	232
Hormones		
Cholecystokinin (P06307)	Small intestine	95
Gastrin (P01350)	Stomach, duodenum, pancreas	80
Promotilin (P12872)	Small intestine(also affects gastric activity)	90
Secretin (P09683)	Duodenum(also affects gastric pH)	103
Somatostatin (P61278)	Stomach, intestine, pancreas	92
Other proteins/peptides involved inthe regulation of specific processesin the digestive tract/immunity		
Gastric inhibitory peptide (P09681)	Stomach	132
Gastric intrinsic factor (P27352)	Stomach	399
Lysozyme C (P61626)	Throughout the gut	130
Dietary proteins		
β-casein, Bovine milk (P02666)	–	209
Gliadin, Wheat (P02863)	–	266
Glutenin, Wheat (P10385)	–	337
Glycinin, Soya (P04347)	–	492
Ovalbumin, Chicken egg (P01012)	–	386
Actin^3^, chicken meat (P60706)	–	375
Myosin^3^, chicken meat (P13538)	–	1939

1 Compiled from the UniProtKB Protein Database [25].

2 The given chain length excludes signal peptide.

3 Initiator methionine not removed from the intact protein sequence (chain length inclusive of the initiator methionine).

### Prediction of the Total Number of Bioactive Peptide Sequences Present in the Intact Gut Endogenous and Dietary Proteins

To predict the number of bioactive peptide sequences encoded within the gut endogenous and dietary proteins, the amino acid sequence of each protein was obtained from an online protein database [25]. The amino acid sequence of each protein was examined for the presence of known bioactive peptide sequences using an online bioactive peptide database [22]. The latter database contained the amino acid sequences of 2609 known bioactive peptides with 48 different bioactivities known to be bioactive based on either *in vitro* or *in vivo* studies [22], [26]. The bioactive peptide database was used according to the instructions given and peptides possessing one or more of the following bioactivities were identified [antiamnestic, angiotensin converting enzyme (ACE)-inhibitor, antithrombotic, stimulating (glucose uptake-, -vasoactive substance release), regulating (ion flow-, stomach mucosal membrane activity-, phosphoinositol mechanism peptide-), antioxidative, bacterial permease ligand, inhibitor (dipeptidyl peptidase IV-, dipeptidyl-aminopeptidase IV-, dipeptidyl carboxypeptidase-, cyclic nucleotide phosphodiesterase 1 (CaMPDE)-, neuropeptide-), hypotensive, activating ubiquitin mediated proteolysis]. The total number of bioactive peptide sequences identified in the intact proteins was recorded for each gut endogenous and dietary protein.

The bioactive peptide frequency (A) and the relative bioactive peptide frequency (Y) are often used to describe the potency of proteins as sources of bioactive peptides [22], [27]. In the present study the frequency of bioactive peptide sequences within the intact protein (A_o_) was calculated as follows:
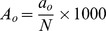
where, a_o_ is the total number of identified bioactive peptides present in the protein or the number of bioactive peptides with a specific activity based on the BIOPEP database [22], N is the total number of amino acid residues within the protein.

The relative frequency of occurrence of bioactive peptides with a specific activity (Yj)[%]:
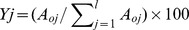
where, A_oj_ is the number of peptides with a specific activity, l is the total number of peptide sequences across all activity categories present within the protein, j is the specified activity.

### Prediction of the Frequency of Potential Bioactive Peptide Sequences Present in the Gastrointestinal Tract After Simulated Digestion in the Stomach, Stomach and Small Intestine and Small Intestine Alone (A_D_)



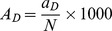
where, a_D_ is the number of identified bioactive peptides present after the simulated (*in silico*) digestion and N is the total number of amino acid residues within the protein.

A prediction of the number of bioactive peptides that would be released from gut endogenous proteins and dietary proteins after upper gastrointestinal tract digestion was made using an *in silico* simulation based on the amino acid sequence of the proteins and the reported specificity of the major proteases present in the gastrointestinal tract. The site of secretion of the gut endogenous proteins was also taken into account. For the gut endogenous proteins secreted in the mouth and stomach and for the dietary proteins, gastric digestion was simulated *in silico* based on the amino acid sequence of the dietary or gut endogenous protein and the specificity of pepsin as documented by Keil [28], [29]. Gastric and small intestinal digestion was predicted based on the specificity of pepsin, trypsin and chymotrypsin as documented by Keil [28], [29]. For endogenous proteins secreted in the small intestine, only small intestinal digestion was simulated taking into account the reported specificity of trypsin and chymotrypsin only. The amino acid sequences of the endogenous and dietary proteins were obtained from a protein sequence database as described above [25]. The *in silico* simulated digestion was conducted using an online Peptide Cutter tool application [28]. The amino acid sequence of each of the predicted resultant peptides for each of the gut endogenous and dietary proteins was then compared to the amino acid sequence of known bioactive peptides using an online bioactive peptide sequence database [22].

## Results

### The Total Number and Frequency (A_O_) of Bioactive Peptide Sequences within the Amino Acid Sequence of Intact Gut Endogenous Proteins and Intact Dietary Proteins

Among the dietary proteins studied, the amino acid chain length of the proteins varied from 209 (β-casein) to 1939 (myosin) amino acids, while for the gut endogenous proteins the range was from 80 (human gastrin) to 5159 (human mucin-2) amino acids (Table 1). The total number of bioactive peptide sequences identified and their corresponding potential bioactivities, within the amino acid sequences of the intact gut endogenous and dietary proteins are shown in Table 2. In addition, the A_O_ values for each activity and for all the activities considered along with Y values for each of the proteins are also shown.

**Table 2:** Number (#) of potential bioactive peptides (per protein molecule) identified in the intact endogenous and dietary proteins and the A_O_ 1 and Y^2^ values.

	Variable	Activity category^3^										OverallA_O_ 4	Total No. ofbioactivepeptides 5
		1	2	3	4	5	6	7	8	9	10		
Endogenous protein													
Mucin-2	#	34	1680	31	80	80	117	7	211	11	19	436	2250
	A_O_	7	326	6	16	16	23	1	41	2	4		
	Y	2	75	1	4	4	5	0	9	0	1		
Mucin-3A	#	12	534	12	87	12	40	0	103	5	8	326	821
	A_O_	5	212	5	35	5	16	0	41	2	3		
	Y	1	65	1	11	1	5	0	13	1	1		
Mucin-3B	#	5	221	7	44	6	9	0	47	3	6	391	352
	A_O_	6	245	8	49	7	10	0	52	3	7		
	Y	1	63	2	13	2	3	0	13	1	2		
Mucin-5AC	#	104	1490	100	111	100	141	5	374	15	31	501	2507
	A_O_	21	298	20	22	20	28	1	75	3	6		
	Y	4	59	4	4	4	6	0	15	1	1		
Mucin-6	#	30	726	33	53	32	96	0	184	8	14	494	1193
	A_O_	12	300	14	25	13	40	0	76	3	6		
	Y	3	61	3	5	3	8	0	15	1	1		
Mucin-7	#	2	124	4	11	2	15	0	68	2	3	654	232
	A_O_	6	349	11	31	6	42	0	192	6	8		
	Y	1	53	2	5	1	6	0	29	1	1		
Mucin-13	#	5	129	5	31	7	20	2	31	3	4	487	240
	A_O_	10	262	10	63	14	41	4	63	6	8		
	Y	2	54	2	13	3	8	1	13	1	2		
Mucin-15	#	0	92	0	18	0	13	0	16	1	0	457	142
	A_O_	0	296	0	58	0	42	0	51	3	0		
	Y	0	65	0	13	0	9	0	11	1	0		
Mucin-20	#	17	207	17	28	18	18	0	46	0	17	541	370
	A_O_	25	303	25	41	26	26	0	67	0	25		
	Y	5	56	5	8	5	5	0	12	0	5		
Serum albumin	#	0	199	0	31	2	37	4	50	6	6	574	339
	A_O_	0	337	0	52	3	63	7	85	10	10		
	Y	0	59	0	9	1	11	1	15	2	2		
Chymotrypsinogen B	#	3	83	4	14	3	9	1	21	2	2	584	143
	A_O_	12	339	16	57	12	37	4	86	8	8		
	Y	2	58	3	10	2	6	1	15	1	1		
Chymotrypsinogen B2	#	2	81	3	14	2	9	1	22	2	2	567	139
	A_O_	8	331	12	57	8	37	4	90	8	8		
	Y	1	58	2	10	1	6	1	16	1	1		
Gastrictriacylglycerol lipase	#	1	138	1	15	1	14	3	32	3	10	586	222
	A_O_	3	364	3	40	3	42	8	84	8	26		
	Y	0	62	0	7	0	7	1	14	1	5		
Pancreaticalpha amylase	#	3	183	3	7	5	26	0	29	4	4	540	268
	A_O_	6	369	6	14	10	52	0	58	8	8		
	Y	1	68	1	3	2	10	0	11	1	1		
Pancreatictriacylglycerol lipase	#	2	167	2	18	2	25	2	26	6	1	563	253
	A_O_	4	372	4	40	4	56	4	58	13	2		
	Y	1	66	1	7	1	10	1	10	2	0		
Pepsin A	#	3	140	2	18	3	20	2	25	2	4	598	223
	A_O_	8	375	5	48	8	54	5	67	5	11		
	Y	1	63	1	8	1	9	1	11	1	2		
Salivary amylase	#	3	184	3	7	5	26	0	28	4	4	540	268
	A_O_	6	371	6	14	10	52	0	56	8	8		
	Y	1	69	1	3	2	10	0	10	1	1		
Trypsin	#	3	81	3	8	5	8	0	14	1	1	539	125
	A_O_	13	349	13	34	22	34	0	60	4	4		
	Y	2	65	2	6	4	6	0	11	1	1		
Cholecystokinin	#	0	38	0	4	1	3	0	9	0	3	611	58
	A_O_	0	400	0	42	11	32	0	95	0	32		
	Y	0	66	0	7	2	5	0	16	0	5		
Gastrin	#	4	33	4	8	4	11	1	9	0	0	950	76
	A_O_	50	413	50	100	50	138	13	113	0	0		
	Y	5	43	5	11	5	14	1	12	0	0		
Promotilin	#	1	32	1	6	1	4	1	10	1	0	644	58
	A_O_	11	356	11	67	11	44	11	111	11	0		
	Y	2	55	2	10	2	7	2	17	2	0		
Secretin	#	3	50	3	7	3	4	0	13	0	1	835	86
	A_O_	29	485	29	68	29	39	0	126	0	10		
	Y	3	58	3	8	3	5	0	15	0	1		
Somatostatin	#	0	27	0	2	0	3	0	9	0	3	500	46
	A_O_	0	293	0	22	0	33	0	98	0	33		
	Y	0	59	0	4	0	7	0	20	0	7		
Gastricinhibitory peptide	#	1	33	2	9	2	2	2	13	0	4	515	68
	A_O_	8	250	15	68	15	15	15	98	0	30		
	Y	1	49	3	13	3	3	3	19	0	6		
Gastricintrinsic factor	#	8	113	8	23	9	15	2	30	2	7	559	223
	A_O_	20	283	20	58	23	38	5	75	5	18		
	Y	4	51	4	10	4	7	1	13	1	3		
Lysozyme C	#	1	45	1	1	2	4	0	8	1	5	538	70
	A_O_	8	346	8	8	15	31	0	62	8	38		
	Y	1	64	1	1	3	6	0	11	1	7		
Dietary protein													
β-casein,Bovine milk	#	10	127	6	15	6	17	1	31	1	0	1105	231
	A_O_	48	608	29	72	29	81	5	148	5	0		
	Y	4	55	3	6	3	7	0	13	0	0		
Gliadin, Wheat	#	1	94	0	11	0	11	0	22	1	1	797	212
	A_O_	4	353	0	41	0	41	0	83	4	4		
	Y	0	44	0	5	0	5	0	10	0	0		
Glutenin, Wheat	#	2	82	0	7	0	7	0	28	1	3	439	148
	A_O_	6	243	0	21	0	21	0	83	3	9		
	Y	1	55	0	5	0	5	0	19	1	2		
Glycinin, Soya	#	2	163	4	22	2	20	0	29	2	3	510	251
	A_O_	4	331	8	45	4	41	0	59	4	6		
	Y	1	65	2	9	1	8	0	12	1	1		
Ovalbumin,Chicken egg	#	1	133	1	22	2	23	0	22	3	3	554	214
	A_O_	3	345	3	57	5	60	0	57	8	0		
	Y	0	62	0	10	1	11	0	10	1	0		
Actin,chicken meat	#	2	149	2	18	3	15	0	28	1	4	597	224
	A_O_	5	397	5	48	8	40	0	75	3	11		
	Y	1	67	1	8	1	7	0	13	0	2		
Myosin,chicken meat	#	1	641	4	112	6	127	37	93	11	23	553	1072
	A_O_	1	331	2	58	3	65	19	48	6	12		
	Y	0	60	0	10	1	12	3	9	1	2		

1 A_O_ is the frequency of occurrence of bioactive fragments in a protein sequence, calculated as 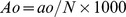 where, a_O_ is the total number of identified bioactive peptides present in the protein or the number of bioactive peptides with a specific activity based on the BIOPEP database [22], N is the total number of amino acid residues within the protein.

2 Y is the relative frequency of occurrence of bioactive fragments with a specific activity in a protein sequence, calculated as 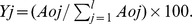

where, Aoj is the number of peptides with a specific activity, l is the total number of peptide sequences across all activity categories present within the protein, j is the specified activity.

3 1 antiamnestic, 2 ACE-inhibitor, 3 antithrombotic, 4 stimulating (glucose uptake-, -vasoactive substance release), 5 regulating (ion flow-, stomach mucosal membrane activity-, phosphoinositol mechanism peptide-), 6 antioxidative, 7 bacterial permease ligand, 8 inhibitor (dipeptidyl peptidase IV inhibitor-, dipeptidyl-aminopeptidase IV inhibitor-, dipeptidyl carboxypeptidase-, CaMPDE-, neuropeptide-), 9 hypotensive, 10 activating ubiquitin mediated proteolysis.

4 Overall A_O_ represents the total number of amino acid sequences corresponding to known bioactive peptides identified per protein molecule across all bioactivity categories normalised for amino acid chain length.

5 The total number of bioactive peptides represents the total number of amino acid sequences corresponding to known bioactive peptides identified per protein molecule across all bioactivity categories (not just the 10 bioactivity categories shown above).

The total number of bioactive peptides, present within the amino acid sequences of the intact protein molecules for the gut endogenous proteins, ranged from 46 peptides for somatostatin to 2507 peptides for Mucin-5AC (Table 2). When based on the subclasses of proteins presented in Table 1, the total number of identified bioactive peptide sequences present within the amino acid sequences of the intact protein molecules ranged from 142–2507 for the mucins, 339 for serum albumin, 125–268 for the digestive enzymes, 46–86 for the hormones and 68–223 for the remaining “other” proteins. For the dietary proteins, the total number of identified bioactive peptide sequences present within the amino acid sequence of the intact proteins ranged from 148 for glutenin to 1072 for myosin.

Among the observed bioactivity categories, angiotensin converting enzyme (ACE)-inhibitory peptide sequences were present in the largest numbers for all of the examined dietary and gut endogenous proteins with Y ranging from 43% for gastrin to 75% for mucin-2 for the gut endogenous proteins and from 44% for gliadin to 67% for actin. For the gut endogenous proteins, the A_O_ for the ACE-inhibitory peptide sequences ranged from 212 for mucin-3A to 485 for secretin while for the dietary proteins A_O_ for the ACE-inhibitory peptide sequences ranged from 243 for glutenin to 608 for β-casein.

In addition to the 10 most abundantly observed bioactive peptide categories presented in Table 2, peptide sequences reportedly possessing other bioactivities were also observed in a few select proteins. For example, opioid peptide sequences were present within the amino acid sequences of all of the dietary proteins but only a few of the endogenous proteins. Similarly, coeliac toxic peptide sequences were present within the amino acid sequences of the wheat proteins gliadin and glutenin only (data not shown).

### Predicted Number and Frequency (A_D_) of Bioactive Peptides Released After Gastric Digestion of Dietary Proteins and Gut Endogenous Proteins Based on an *in silico* Simulation

The number of bioactive peptides (and their corresponding predicted bioactivities) predicted to be released after gastric digestion of gut endogenous proteins secreted in the mouth and stomach and of dietary proteins based on an *in silico* simulation of gastric digestion are presented in Table 3. The total number of bioactive peptides predicted to be released after gastric digestion of the gut endogenous proteins ranged from 0 to 12 bioactive peptides per protein molecule for lysozyme C and serum albumin respectively. When grouped into the protein subclasses shown in Table 1, the total number of predicted bioactive peptides after digestion was 1–11 peptides per molecule for the mucins, 12 for serum albumin, 2–8 for the digestive enzymes, 0–2 for the hormones and 0–4 for the “other” proteins. For the dietary proteins, between 1 (glutenin and gliadin) and 11 (myosin) bioactive peptides were predicted to be released per protein molecule after gastric digestion. When the number of predicted bioactive peptides was presented in relation to the number of amino acids in each protein, the A_D_ value for the mucins, serum albumin, digestive enzymes, hormones and “other” proteins was 1–6, 20, 4–21, 0–22 and 0–10 respectively. For the dietary proteins, the A_D_ value ranged from 3 for (glutenin and actin) to 14 for (β-casein).

**Table 3:** Number of potential bioactive peptides (per protein molecule) predicted to be released and A_D_ 1 value after gastric digestion of both gut endogenous proteins secreted in the mouth and stomach and selected dietary proteins based on an *in silico* digestion model.

Protein	Activity^2^					OverallA_D_	Total No. ofpredicted bioactivepeptides released^3^^,^ 4
	2	4	6	8	10		
Endogenous protein							
Mucin-5AC	7	1				2	8
Mucin-6	4	5	2	3		5	11
Mucin-7		1	1	1		6	2
Mucin-13	1					2	1
Mucin-20	1					1	1
Serum albumin	8	3	1	3		20	12
Gastric triacylglycerol lipase	4	3		3	1	21	8
Pepsin A		1	1	1		5	2
Salivary amylase	1	1				4	2
Gastrin			1			13	1
Promotilin	2					22	2
Secretin						0	0
Somatostatin			1			11	1
Gastric inhibitory peptide		1		1		8	1
Gastric intrinsic factor	2	1		2		10	4
Lysozyme C						0	0
Dietary protein							
β-casein, Bovine milk	2	1	1	1		14	3
Gliadin, Wheat	1			1	1	4	1
Glutenin, Wheat	1					3	1
Glycinin, Soya	4					8	4
Ovalbumin, Chicken egg	4	1		1		13	5
Actin, chicken meat	1					3	1
Myosin, chicken meat	8	3		4	1	6	11

1 A_D_ is the frequency of occurrence of bioactive peptides after digestion of the protein, calculated as 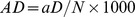 where, a_D_ is the number of identified bioactive peptides present after the simulated (*in silico*) digestion and N is the total number of amino acid residues within the protein.

2 2 ACE-inhibitor, 4 stimulating (glucose uptake-, -vasoactive substance release), 6 antioxidative, 8 inhibitor (dipeptidyl peptidase IV inhibitor-, dipeptidyl-aminopeptidase IV inhibitor-, dipeptidyl carboxypeptidase-, CaMPDE-, neuropeptide-), 10 activating ubiquitin mediated proteolysis.

3 The total number of peptides released is a summation of all the bioactive peptides predicted to be released after digestion of the intact proteins.

4 Some of the predicted bioactive peptides have more than one activity. Hence, the total number of bioactive peptides released may be less than the summation of the number of bioactive peptides from the individual activity categories.

Bioactive peptides with ACE-inhibitory activity were predicted to be present after gastric digestion in higher numbers compared to peptides in the other activity categories with a total of 51 ACE-inhibitory peptides predicted to be present post-digestion across all of the examined proteins as compared to 0–22 predicted peptides for all of the other activity categories. Serum albumin and myosin were predicted to yield the largest number of ACE-inhibitory peptides after peptic digestion with 8 ACE-inhibitory peptides per protein molecule. This was closely followed by 7 ACE-inhibitory peptides for mucin-5AC. Considerably fewer ACE-inhibitory peptides were predicted (0–4 peptides per molecule) for the remaining gut endogenous and dietary proteins. The other predicted bioactivities with identified peptides were stimulating (glucose uptake-), inhibitor (dipeptidyl peptidase IV-, dipeptidyl-aminopeptidase IV-), and antioxidative activities and activation of ubiquitin mediated proteolysis.

### Predicted Number and Frequency (A_D_) of Bioactive Peptides Released After Gastric and Small Intestinal Digestion of Dietary Proteins and Gut Endogenous Proteins Based on an *in silico* Simulation

The total number of bioactive peptides predicted to be released after gastric and small intestinal digestion *in silico* for the gut endogenous proteins secreted into the mouth and stomach, and that therefore underwent digestion in the stomach and small intestine, ranged from 1 peptide per protein molecule for secretin to 39 peptides per protein molecule for mucin-5AC (Table 4). When the proteins were divided into subclasses based on their functions as shown in Table 1, the predicted bioactive peptides released per protein molecule were 2–39 for the mucins, 22 for serum albumin, 4–15 for the digestive enzymes, 1–5 for the hormones and 3–10 for the “other” proteins. For the dietary proteins, the predicted number of bioactive peptides released after digestion (*in silico*) ranged from 1 for gliadin to 55 for myosin. When the size of the proteins were taken into account, the predicted A_D_ value for the mucins, serum albumin, digestive enzymes, hormones and “other” proteins was 3–17, 37, 11–40, 10–56 and 23–31 respectively. For the dietary proteins, the predicted A_D_ value ranged from 4 for gliadin to 38 for β-casein.

**Table 4:** Number of potential bioactive peptides (per protein molecule) predicted to be released and A_D_ 1 value after gastric plus small intestinal digestion for gut endogenous proteins secreted in the mouth and stomach and selected dietary proteins based on an *in silico* digestion model.

Protein	Activity^2^							A_D_	Total No. of predicted bioactivepeptides released^3^^,^ 4
	2	4	5	6		8	9		10		
Endogenous protein											
Mucin-5AC	38			3						8	39
Mucin-6	17	4		5		3				9	22
Mucin-7	2	2		3		3	1			17	6
Mucin-13	6									12	6
Mucin-20	2									3	2
Serum albumin	17	3		4		2				37	22
Gastric triacylglycerol lipase	10	3		2		3				40	15
Pepsin A	3	1		1		3	1			11	4
Salivary amylase	8	2	1	3						22	11
Gastrin	2			1						38	3
Promotilin	4			1						56	5
Secretin	1									10	1
Somatostatin	1			1						22	2
Gastric inhibitory peptide	1	1				1				23	3
Gastric intrinsic factor	6	1		2		2				25	10
Lysozyme C	3			1						31	4
Dietary protein											
β-casein, Bovine milk	8	1		1		1				38	8
Gliadin, Wheat	1									4	1
Glutenin, Wheat	2			1						9	3
Glycinin, Soya	10			4		3	1			30	15
Ovalbumin, Chicken egg	7	1		3		1				23	9
Actin, chicken meat	7			1		1				24	9
Myosin, chicken meat	49	4	1	4		5	1		1	28	55

1 A_D_ value is the frequency of occurrence of bioactive peptides after digestion of the protein, calculated as 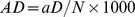 where, a_D_ is the number of identified bioactive peptides present after the simulated (*in silico*) digestion and N is the total number of amino acid residues within the protein.

2 2 ACE-inhibitor, 4 stimulating (glucose uptake-, -vasoactive substance release), 5 regulating (ion flow-, stomach mucosal membrane activity-), 6 antioxidative, 8 inhibitor (dipeptidyl peptidase IV inhibitor-, dipeptidyl-aminopeptidase IV inhibitor-, dipeptidyl carboxypeptidase-, CaMPDE-, neuropeptide-), 9 hypotensive, 10 activating ubiquitin mediated proteolysis.

3 The total number of peptides released is a summation of all the bioactive peptides predicted to be released after digestion of the intact proteins.

4 Some of the predicted bioactive peptides have more than one activity. Hence the total number of bioactive peptides released may be less than the summation of the number of bioactive peptides from the individual activity categories.

After *in silico* simulated gastric and small intestinal digestion, the most abundant bioactive peptides predicted to be present were the ACE-inhibitory peptides ranging from 1 peptide per protein molecule for secretin, somatostatin, gastric inhibitory peptide and gliadin to 38 for mucin-5AC. Other gut endogenous proteins from which notable amounts of ACE-inhibitory peptides were predicted to be released were mucin-6 (17 peptides per molecule), serum albumin (17 peptides per molecule) and gastric triacylglycerol lipase (10 peptides per molecule). Among the food proteins evaluated, myosin was predicted to yield the greatest number of ACE-inhibitory peptides (49 peptides per molecule). Other bioactive peptides predicted to be present after gastric and small intestinal digestion (based on an *in silico* simulation) across all proteins were glucose uptake-or vasoactive substance release-stimulating (0–4 per molecule), dipeptidyl peptidase IV- or dipeptidyl-aminopeptidase IV-inhibitor (0–5 peptides per molecule), antioxidative (0–5 peptides per molecule), ion flow- or stomach mucosal membrane activity- regulating (0–1 peptides per molecule), and hypotensive (0–1 peptides per molecule) peptides and peptides activating ubiquitin mediated proteolysis (0–1 peptides per molecule).

### Predicted Number and Frequency (A_D_) of Bioactive Peptide Sequences Released After Small Intestinal Digestion of Gut Endogenous Proteins Secreted in the Small Intestine Based on an *in silico* Simulation

For endogenous gut proteins that are secreted into the small intestine (for example, the pancreatic enzymes and small intestinal mucins) and therefore would not be subject to digestion in the stomach, an *in silico* analysis of the bioactive peptides that would be predicted to be released after intestinal digestion alone was performed (Table 5). For these proteins mucin-2, serum albumin and pancreatic amylase had the greatest predicted numbers of bioactive peptides released, with 24, 14 and 14 bioactive peptides respectively per molecule; while secretin had the least (1 peptide per molecule). Within the subclasses of proteins based on protein function and presented in Table 1, the predicted number of bioactive peptides released after digestion was 2–24 peptides per molecule for the mucins, 14 peptides per molecule for serum albumin, 4–14 peptides per molecule for the digestive enzymes and 1–2 peptides per molecule for the hormones and 2 peptides per molecule for lysozyme C. The corresponding A_D_ values were 5–10 for the mucins, 24 for serum albumin, 13–28 for the digestive enzymes and 10–25 for the hormones and 15 for lysozyme C.

**Table 5:** Number of potential bioactive peptides (per protein molecule) predicted to be released and A_D_ 1 value after small intestinal digestion for gut endogenous proteins secreted in the small intestine based on an *in silico* digestion model.

Protein	Activity^2^					A_D_	Total No. of predicted bioactivepeptides released^3^^,^ 4
	2	5	6	8	9		
Endogenous protein							
Mucin-2	18	2	4	4	4	5	24
Mucin-3A	12					5	12
Mucin-3B	9					10	9
Mucin-13	3					6	3
Mucin-15	1		2			6	2
Mucin-20	4					6	4
Serum albumin	9		6	1	1	24	14
Chymotrypsinogen B	5					20	5
Chymotrypsinogen B2	5					20	5
Pancreatic amylase	12	1	5			28	14
Pancreatic triacylglycerol lipase^5^	5					13	6
Trypsin	3		1			17	4
Cholecystokinin	1	1				21	2
Gastrin	2					25	2
Promotilin	1		1			22	2
Secretin	1					10	1
Somatostatin	2					22	2
Lysozyme C	2					15	2

1 A_D_ value is the frequency of occurrence of bioactive peptides after digestion of the protein, calculated as 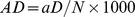 where, a_D_ is the number of identified bioactive peptides present after the simulated (*in silico*) digestion and N is the total number of amino acid residues within the protein.

2 2 ACE-inhibitor, 5 regulating (ion flow-, stomach mucosal membrane activity-, phosphoinositol mechanism peptide-), 6 antioxidative, 8 inhibitor (dipeptidyl peptidase IV inhibitor-, dipeptidyl-aminopeptidase IV inhibitor-, dipeptidyl carboxypeptidase-, CaMPDE-, neuropeptide-), 9 hypotensive.

3 The total number of peptides released is a summation of all the bioactive peptides predicted to be released after digestion of the intact proteins.

4 Some of the predicted bioactive peptides have more than one activity. Hence the total number of bioactive peptides released may be less than the summation of the number of bioactive peptides from the individual activity categories.

5 Pancreatic triacylglycerol is predicted to release 1 immunostimulating peptide. Peptide not shown in the table, but is reflected in the corresponding total number of predicted bioactive peptides released.

## Discussion

All of the protein amino acid sequences were sourced from the UniProt Protein Knowledgebase, a standard repository of protein sequences related information [25]. BIOPEP, the database of bioactive peptides used in the present study, is a widely recognised and utilised tool for the bioinformatics based prediction of bioactive peptides in a given amino acid sequence [22], [27], [30], [31]. The associated bioactivity of the peptide sequences listed in the BIOPEP database is documented and continually updated based on previous and on-going *in vitro* and *in vivo* studies [22], [26], [32], [33]. The resultant peptides generated after simulated gastrointestinal digestion were predicted using Peptide Cutter, an enzymatic cleavage prediction software [28], that is hosted by the ExPASY server, a standard tool used in bioinformatics and mass spectrometry-based studies [34].

The findings of the present study are based on an *in silico* gastrointestinal digestion prediction-model. The model is based on the amino acid sequence (primary structure) of the intact proteins and knowledge about the specificity of proteases in the gastrointestinal tract. Being an *in silico* model, it cannot be concluded with certainty that the purported bioactive peptides will be generated after the actual *in vivo* gastrointestinal tract digestion of gut endogenous proteins. However, there are similarities between data generated in the presently reported study and data generated in other *in silico, in vitro* and *in vivo* studies. For example, in the present study β-casein was found to be the greatest potential source of bioactive peptides, including ACE-inhibitory peptides. This finding is consistent with another *in silico* study that examined a range of food proteins and predicted that bovine caseins were the greatest source of ACE-inhibitory peptides [35]. In addition, Boutrou et al [11] investigated the kinetics of the release of peptides from either casein or whey proteins in the jejunum of humans, and reported that β-casein released both larger numbers of bioactive peptide fragments and generated peptides with a diverse range of bioactivities. Moreover, and in line with our own findings, *in vitro* studies have shown that the antihypertensive peptides VPP and IPP present in the amino acid sequence of bovine β-casein, which are known to be released during lactobacilli-based fermentation of milk [36], are not released during enzymatic digestion using an *in vitro* digestion model that simulated digestion in the gastrointestinal tract [37].

Overall, the *in silico* technique used in the presently reported study does demonstrate that large numbers of bioactive peptide sequences do exist within the amino acid sequences of endogenous proteins that may be cleavable by the digestive enzymes and it is likely that in the process of digestion within the gut, bioactive peptides would be liberated from the gut endogenous proteins, particularly given that it is known from *in vivo* studies that as much as 80% of the endogenous protein secreted into the gastrointestinal tract is digested and reabsorbed [14], [15]. The presently reported study does not include analysis of two major contributors to the non-dietary nitrogenous losses in the gut, namely, bacterial proteins and the sloughed epithelial cells. Also factors that may influence *in vivo* protein digestion in the gastrointestinal tract, such as, the tertiary structure of the proteins, the effects of food processing on protein digestion, and the influence of bacterial enzymatic digestion have not been taken into account. An attempt has been made, however, to analyse a range of gut endogenous proteins secreted at different sites within the gut and with known amino acid sequences.

All of the dietary and gut endogenous proteins evaluated in the present study contained large numbers of peptide sequences within the greater amino acid sequence of the intact protein that corresponded to the sequences of known bioactive peptides, at least based on the BIOPEP bioactive peptide database [22]. Furthermore, the total number of bioactive peptide sequences present in the overall amino acid sequence of the intact proteins varied across both dietary and gut endogenous proteins, although the range was much greater for the endogenous proteins. The mucin proteins generally contained the greatest number of bioactive peptide sequences while the hormone molecules contained the least. In comparison with the dietary proteins, 16 of the 26 gut endogenous proteins contained a similar or greater number of bioactive peptide sequences per molecule. This suggests that based on amino acid sequence, the gut endogenous proteins may contain quantitatively significant amounts of bioactive peptides. In general, for both the food and gut endogenous proteins, smaller proteins contained comparatively fewer bioactive peptide sequences when compared to the larger proteins. The latter observation indicated, not unexpectedly, that the longer the amino acid chain of a protein, the higher the probability of finding peptide sequences that correspond to previously studied and reported bioactive peptides documented in the BIOPEP database [22].

If the gut endogenous proteins and food proteins are considered in terms of the potential bioactive profile (the relative number of bioactive peptide sequences within each bioactivity category), both gut endogenous and dietary proteins were similar with ACE-inhibitory peptide sequences being present in the greatest numbers. This may be attributed to the fact that ACE-inhibitory peptides have been researched more extensively in comparison to all of the other bioactivities and hence the bioactive peptide database used in the present study contains a much higher proportion of known ACE-inhibitory peptides as compared to bioactive peptides with other activities [2], [38]. Both the gut endogenous proteins and the dietary proteins seem to contain remarkably similar relative numbers of bioactive peptides within each activity category particularly given the very different amino acid sequences across the different proteins. For example, ACE-inhibitory peptides comprised 43–75% of the total number of bioactive peptides found across the proteins while inhibitor peptides comprised 10–29%, antioxidative peptides comprised 3–14%, stimulating peptides comprised 3–13% and hypotensive peptides comprised 0–2%. Overall, large numbers of bioactive peptide sequences were observed in the intact gut endogenous protein amino acid sequences. In comparison to the dietary proteins examined in the present study, gut endogenous proteins were similar in terms of being a potential source of bioactive peptides.

Significant numbers of bioactive peptides were predicted to be released after gastric digestion (based on an *in silico* digestion model) of both food and gut endogenous proteins; however the numbers predicted were only 0–3.5% (average across all examined proteins = 1.0%) of the total number of bioactive peptide amino acid sequences identified in the intact amino acid sequences of each protein. It would appear, based on the *in silico* prediction used in the present study, that for both the dietary and gut endogenous proteins most of the predicted bioactive peptide sequences present in the intact proteins would not be released during gastric enzymic digestion. In terms of the bioactive peptides that were predicted to be released after gastric digestion, the gut endogenous proteins appeared to be similar to the dietary proteins both in terms of the total number of predicted bioactive peptides and the number of predicted bioactive peptides normalised for the amino acid chain length of the protein (A_D_ values).

The number of bioactive peptides predicted to be released after gastric and small intestinal digestion combined were considerably higher compared to gastric digestion alone but were still much fewer in comparison to the number of bioactive peptide amino acid sequences identified within the intact protein (3.3% of the total number of the identified bioactive peptides were predicted to be released across protein sources). It was predicted that after combined gastric and small intestinal digestion, many endogenous proteins were an equal source of bioactive peptides compared to the selected dietary proteins with a mean A_D_ across all of the endogenous proteins of 23 compared to 22 for the dietary proteins. Moreover at least two of the endogenous proteins had a greater A_D_ value in comparison with β-casein, a known rich source of bioactive peptides.

Not all gut endogenous proteins are secreted ubiquitously throughout the gastrointestinal tract [25]. For example while serum albumin is known to be secreted into both the stomach and the small intestine [39], [40], trypsin is only secreted into the duodenum and therefore is only subject to digestion in the small intestine. For proteins that are secreted in the small intestine, digestion in the gastrointestinal tract was predicted based on an *in silico* model for small intestinal digestion alone with the two major intestinal enzymes trypsin and chymotrypsin. The number of bioactive peptides predicted to be present after small intestinal digestion alone were much fewer in comparison to those predicted after both gastric and intestinal digestion. For example, the total number of bioactive peptides predicted to be released after small intestinal digestion of serum albumin (14 bioactive peptides per protein molecule) was much lower than that predicted for gastric and intestinal digestion (22 bioactive peptides per protein molecule). Despite this, the results of the present study would predict that gut endogenous proteins secreted into the small intestine also appear to be significant sources of bioactive peptides.

After small intestinal digestion alone, the predicted released bioactive peptides possessed fewer bioactivities. For example, across all of the examined proteins (gut endogenous and dietary) the bioactive peptides predicted to be released after gastric and intestinal digestion had collectively up to 7 different bioactivities, while after small intestinal digestion alone, the predicted bioactive peptides collectively had only up to 3 different bioactivities, with an exception of serum albumin and mucin-2 which were predicted to release bioactive peptides in two additional bioactivity categories. Furthermore, for proteins that are secreted in both the stomach and small intestine, the same protein was predicted to release different bioactive peptide sequences in terms of total number and amino acid sequence depending on the site of digestion (gastric+small intestinal vs. small intestinal alone; Table 6).

**Table 6:** Amino acid^1^ sequences of bioactive peptides predicted to be released after mouth to ileum digestion of selected proteins based on an *in silico* digestion model.

Protein	Bioactivity^2^	Gastric	Gastric+Small intestinal	Small intestinal
Gut endogenous				
Serum albumin				
	2	KA, IA, LF, QK, GM,RL, VE, AA	IA, LF, PR, LY, QK, AW,EK, GM, EY, AR, EK, KP,VR, VPK, VE, VK, AA	LF, LY, AW, NY, EY, AR,VF, VPK, TF
	4	LV, LL	LV	
	6	LHT	LHT, KP	LK, LY, TY
	8	KA, LL		EF
	9			EF
Somatostatin				
	2	AA	AA, QK	NF, TF
	6	EL	EL	
Dietary				
β-casein, bovine milk				
	2	HL, PLP	AR, VK, HK, EMPFPK, HL, PLP, GPFPIIV	
	4	LI, LL	LI, LL	
	6	HL	HL	
	8	VA, LL	VA, LL	
Ovalbumin, Chicken egg				
	2	KE, YAEERYPIL, KG, EK	LY, LW, EK, VY, PR, GR	
	4	LL	LL	
	6		LK, LY, VY	
	8	LL	LL	

1 All amino acids are denoted using ‘the one-letter notation for amino acid sequences’ from the International Union of Pure and Applied Biochemistry and International Union of Biochemistry, 1971: Alanine = A; Arginine = R; Asparagine = N; Aspartic acid = D; Cysteine = C; Glutamic acid = E; Glutamine = Q; Glutamine or Glutamic acid = Z; Glycine = G; Histidine = H; Isoleucine = I; Leucine = L; Lysine = K; Methionine = M; Phenylalanine = F; Proline = P; Serine = S; Threonine = T; Tryptophan = W; Tyrosine = Y; Valine = V.

2 2 ACE-inhibitor, 4 stimulating (glucose uptake-, -vasoactive substance release), 6- antioxidative, 8 inhibitor (dipeptidyl peptidase IV inhibitor-, dipeptidyl-aminopeptidase IV inhibitor-, dipeptidyl carboxypeptidase-, CaMPDE-, neuropeptide-), 9 hypotensive.

For the most abundantly predicted bioactivity, ACE-inhibition, based on the present *in silico* digestion model, it would appear that on a per molecule basis, gut endogenous proteins may be similar to dietary proteins in terms of the potential to release ACE-inhibitory peptides in the upper gastrointestinal tract as a result of digestion.

The majority of the bioactive peptide sequences present in the amino acid sequence of the intact gut endogenous protein and after “*in silico”* digestion were di- or tri- peptides, while for the dietary proteins, bioactive peptides of 6 to 9 amino acids in length were also observed (Table 6). The 3 known opioid agonist peptides in β-casein (5 to 11 amino acid long, data not shown) were also longer in chain length than the average bioactive peptide chain length observed in the gut endogenous proteins. In terms of the amino acid composition of the gut endogenous proteins evaluated, it is of note that, many of them contain significant amounts of glycine or proline or both, and it has been reported that a high content of glycine and proline is related to a higher probability of finding bioactive peptide fragments [32].

This study makes no attempt to investigate the efficacy of bioactive peptides but rather provides an *in silico* prediction of the number and types of bioactive peptides that potentially can be generated in the gastrointestinal tract during digestion.

To put the current findings into context an attempt was made to predict the amounts of bioactive peptides that may be released into the gastrointestinal tract per day from either dietary protein or gut endogenous protein sources (Table 7). For the daily dietary protein intake, the food proteins examined in the presently reported study were used as ingredients for a theoretical diet. This model diet was formulated to contain approximately 40 g of protein which represents the Food and Agricultural Organisation of the United Nations’ (FAO) recommended daily protein intake for a healthy adult weighing 60 kg [1]. The proportion of each individual dietary protein was derived based on a model diet assumed to contain 127 g of dairy products, 128 g of wheat-based products, 25 g of soya products, 44 g (1, medium sized) egg and 46 g of roasted chicken (the vegetables, fruits, fats and sugars in the diet were omitted from the present estimations as they contain negligible amounts of proteins). The amount of endogenous protein secreted into the gastrointestinal tract was estimated based on the reported amounts of gut endogenous protein nitrogen secreted into the gastrointestinal tract, but excludes protein nitrogen derived from epithelial and bacterial cells [41]. Based on the model diet, it is predicted that in a healthy adult, dietary proteins may contribute 1842 mg, while the gut endogenous proteins (excluding microbial protein and sloughed cells) may yield up to 2689 mg of bioactive peptides per day. Given that microbial protein and sloughed cells, which make up approximately two thirds of the total gut non-dietary protein, were not included in the latter prediction it is likely that the amount of bioactive peptides derived from gut endogenous proteins would be much higher.

**Table 7:** Predicted quantity of bioactive peptides (mg/d) released after digestion of either dietary proteins or gut endogenous proteins in the gastrointestinal tract.

	Predicted mean quantity of bioactive peptides released^1^ (mg/g protein)	Estimated quantity of bioactive peptides released after digestion in the gastrointestinal tract(mg/d)	
		Protein source^2^	
**Dietary protein**			
β-casein, bovine milk	87	Dairy	348
Gliadin and Glutenin, wheat	14	Wheat products	196
Glycinin, soya	69	Soya products	207
Ovalbumin, chicken egg	54	Chicken egg products	324
Actin and Myosin, chicken meat	59	Chicken meat	767
		**Predicted (total) amount of bioactive peptides (mg/d)**	**1842**
**Gut endogenous protein^3^**			
Mucin, Serum albumin, Pepsin A, Gastrin and Lysozyme C	56	**Predicted (total) amount of bioactive peptides (mg/d)**	**2689**

1 Estimated based on the predicted total number of bioactive peptides released after gastric and small intestinal digestion (from Table 4), and the moles and molar masses of the respective proteins; and considering that the majority of the predicted bioactive peptides are ‘dipeptides’. All of the evaluated food proteins are used as a model for the remaining proteins in the respective food product.

2 The model diet is based on a recommended diet for a healthy adult weighing 60 kg, supplying 0.66 g/kg body weight protein per day, amounting to a protein intake of 40 g per day, designed to comply with the FAO recommendations 1]; whereby dairy, wheat, soya products, chicken egg products, chicken meat contribute 4, 14, 3, 6 and 13 g of protein respectively; Protein content of food products estimated based on the United States Department of Agriculture (USDA) Nutrient Data Laboratory database 42].

3 Calculated based on Moughan, 2011 41], using the amount of gut endogenous protein nitrogen secreted into the gastrointestinal tract, but, excludes protein nitrogen derived from epithelial and bacterial cells.

In conclusion, based on an *in silico* prediction it would appear that gut endogenous proteins may be an important and diverse source of bioactive peptides, in comparison with food proteins, particularly given that gut endogenous proteins are likely to be present in the gastrointestinal tract at a more constant concentration and composition than proteins derived from the diet. However, further *in vitro* and *in vivo* work is needed to corroborate the *in silico* predictions of the present study.
